# Distinctive origin and evolution of endemic thistle of Korean volcanic island: Structural organization and phylogenetic relationships with complete chloroplast genome

**DOI:** 10.1371/journal.pone.0277471

**Published:** 2023-03-13

**Authors:** Bongsang Kim, Yujung Lee, Bomin Koh, So Yun Jhang, Chul Hee Lee, Soonok Kim, Won-Jae Chi, Seoae Cho, Heebal Kim, Jaewoong Yu

**Affiliations:** 1 Department of Agricultural Biotechnology and Research Institute of Agriculture and Life Sciences, Seoul National University, Seoul, Republic of Korea; 2 eGnome, Inc, Seoul, Republic of Korea; 3 Interdisciplinary Program in Bioinformatics, Seoul National University, Seoul, Republic of Korea; 4 County Office of Ulleung-gun, Gyeongsangbuk-do, Korea; 5 Microorganism Resources Division, National Institute of Biological Resources, Incheon, Republic of Korea; National Cheng Kung University, TAIWAN

## Abstract

Unlike other *Cirsium* in Korea, *Cirsium nipponicum* (Island thistle) is distributed only on Ulleung Island, a volcanic island off the east coast of the Korean Peninsula, and a unique thistle with none or very small thorns. Although many researchers have questioned the origin and evolution of *C*. *nipponicum*, there is not much genomic information to estimate it. We thus assembled the complete chloroplast of *C*. *nipponicum* and reconstructed the phylogenetic relationships within the genus *Cirsium*. The chloroplast genome was 152,586 bp, encoding 133 genes consisting of 8 rRNA genes, 37 tRNA genes, and 88 protein-coding genes. We found 833 polymorphic sites and eight highly variable regions in chloroplast genomes of six *Cirsium* species by calculating nucleotide diversity, as well as 18 specific variable regions distinguished *C*. *nipponicum* from other *Cirsium*. As a result of phylogenetic analysis, *C*. *nipponicum* was closer to *C*. *arvense* and *C*. *vulgare* than native *Cirsium* in Korea: *C*. *rhinoceros* and *C*. *japonicum*. These results indicate that *C*. *nipponicum* is likely introduced through the north Eurasian root, not the mainland, and evolved independently in Ulleung Island. This study contributes to further understanding the evolutionary process and the biodiversity conservation of *C*. *nipponicum* on Ulleung Island.

## Introduction

*Cirsium nipponicum* (Maxim.) Makino is a perennial flowering plant that can be found near the seashore and belongs to the Carduoideae subfamily in Asteraceae. Among eight *Cirsium* species that grow naturally in Korea [1], *C*. *nipponicum*, also known as island thistle, is predominantly found only on Ulleung Island, an oceanic volcanic island on the east coast of the Korean Peninsula, and has no or very small thorns on its leaves. Like other *Cirsium* species traditionally used as a medicinal plant in East Asia for their bioactivities, including hepatoprotective, antioxidant, and antidiabetic activities [2–7], dried *C*. *nipponicum* has also been used as a medicinal source. It is an abundant producer of polyphenols and flavonoids such as cirsimarin and pectolinarin with antioxidant and anti-inflammatory activity [3, 8, 9]. In addition, the leaves known to be different from other *Cirsium* are also used as a resource for vegetables. Based on the fact that other Caruoideae species like milk thistle, were studied to investigate medicinal effects [10–12], studies were also conducted on *C*. *nipponicum* [3, 8, 13, 14].

**Fig 1:**
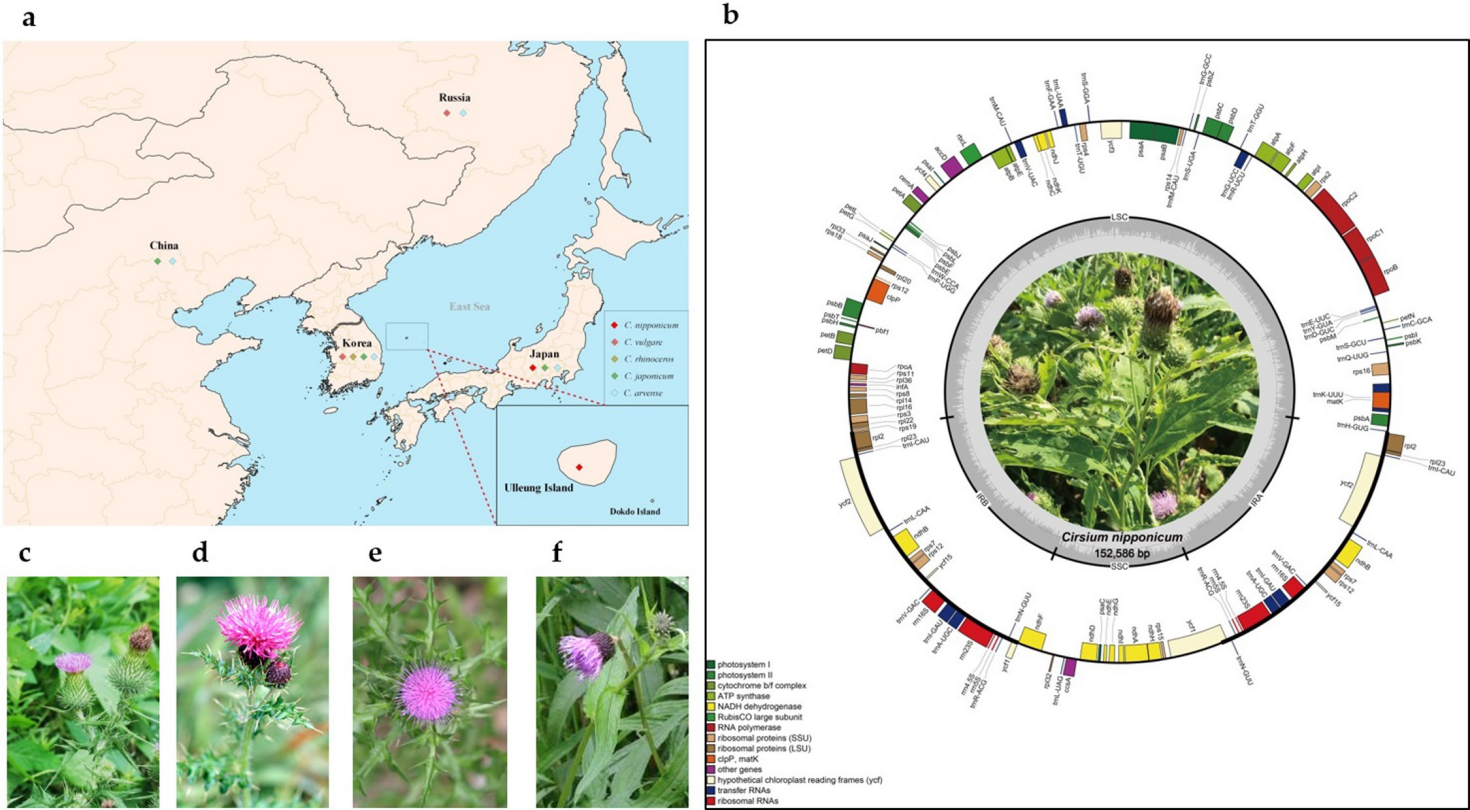
*Cirsium* species distribution map and chloroplast genetic map. (a) Geographical distribution of *Cirsium* species around Korea (source: Natural Earth). (b) Genetic map of the *C*. *nipponicum*. Genes drawn outside the circle are transcribed counterclockwise, and others inside are transcribed clockwise. (c) *C*. *vulgare* distributed near Ulleung Island, provided by Bio Resource Information Service (BRIS). (d) *C*. *rhinoceros* distributed near Ulleung Island, provided. (e) *C*. *japonicum* distributed near Ulleung Island, provided by National Institute of Biological Resources (NIBR). (f) *C*. *arvense* distributed near Ulleung Island, provided by NIBR.

Although several *Cirsium* species are distributed in Korea and neighboring countries (Fig 1A), the origin of the Korean *C*. *nipponicum*, which is distributed only on Ulleung Island, is not yet clear. Previous studies on phylogenetic relationships have shown that *C*. *nipponicum* is distinct from other endemic *Cirsium* [1, 15]. However, there is a limitation to understanding the biological differences based on genomic studies among *Cirsium* species, as few studies have been conducted using the DNA of *C*. *nipponicum* in recent decades. Furthermore, despite the presence of other comparative analyses with *C*. *nipponicum*, the phylogenetic analyses have also been performed in a limited way using combinations of morphological characteristics and only small portions of genomic DNA, such as DNA barcode regions, which are problematic even in the evolutionary process [16, 17].

Islands are considered a prosperous region in terms of plant species diversity, and Ulleung Island is one of the biodiversity hot spots in Korea [18, 19]. Nonetheless, the current biological species in islands are under threat from the loss of native habitats and climate change, such that many plants in Ulleung Island are suffering from various forms of development [20–22]. Under these circumstances, conservation work on endemic species of Ulleung Island, including *C*. *nipponicum*, has just begun, and at the same time, genome construction of these species is required. Since the development of next-generation sequencing [23] technology has enabled researchers to study and understand the genome from a broader and deeper perspective, the acquisition of genetic resources has been activated and the quality has also improved. In addition, many projects involving genomic data, such as genome skimming or DNA barcoding, have been accompanied. Therefore, we aimed to present the chloroplast genomic data of *C*. *nipponicum* based on future studies, as genomic data can complement small remaining challenges and provide an accurate method for the biological understanding and biodiversity of Ulleung Island.

Plastid genomes were sequenced before the nuclear genome in most plant organisms because of their conserved traits, such as gene contents, low recombination, self-replication, genome structure, small compact size, maternal inheritance, and moderate substitution rates for comparative analysis within related species [24–26]. For those reasons, the study of the chloroplast genome is regarded as a valuable resource for investigating phylogenetic analysis, population genetics, or plant systematics. For example, previous studies using the chloroplast genome have inferred phylogenetic relationships in traditionally intricate groups of tribe Cardueae [27, 28]. Moreover, variable regions such as repeat sequences or intergenic spacer (IGS) in chloroplast genomes of many species have been explored as helpful information for effective strategies to conserve endangered species [29]. Hence, constructing the chloroplast genome of *C*. *nipponicum* will be of great help in studying the evolutionary process of *Cirsium* and its adaptation to specific environments.

In this study, we assembled a complete chloroplast genome of *C*. *nipponicum* for the first time through NGS paired-end data and compared its chloroplast genome with other previously published chloroplast genomes. Then, we identified the genetic structure of the *C*. *nipponicum* chloroplast genome and performed comparative analyses with other *Cirsium* species. As a result, repeat elements and highly variable regions within *Cirsium* species were detected to distinguish *C*. *nipponicum* from others and constructed phylogenetic trees to observe the evolutionary relationship among Carduoideae.

## Methods

### Plant material, DNA extraction, and sequencing

Fresh leaves of *C*. *nipponicum* were collected from a conservation garden in Ulleunggun Agriculture Technology Center, Ulleung-gun, Gyeonsangbuk-do, Korea (37°27’37.0"N 130°52’29.9"E) under guide of Chul Hee Lee (research officer of Ulleunggun Agriculture Technology Center). The plant materials produced and used in this study comply with Korean guidelines and legislation. All the experiments were carried in accordance with national and international guidelines. Genomic DNA of *C*. *nipponicum* was extracted from leaf tissues using a cetyl trimethylammonium bromide (CTAB)-based protocol [30]. A paired-end library with a 2 x 151 paired-end (PE) was constructed following the manufacturer’s instructions (Illumina, USA) and sequenced using HiSeq platform.

### Read data processing and chloroplast genome assembly

Quality control of removing low-quality reads and adaptor sequences was performed using fastQC and Trimmomatic programs [31, 32]. The adapter sequences were removed, and the end of reads with Phred score less than 20 was trimmed. Afterward, high-quality reads were assembled using GetOrganelle-1.7.1 [33], and then annotated using PGA v3 and GeSeq based on the four reference chloroplast genomes: *C*. *rhinoceros* (NC_044423.1), *C*. *eriophorum* (NC_036966.1), *C*. *vulgarae* (NC_036967.1), and *C*. *arvense* (NC_036965.1) [34, 35]. The tRNA genes were verified with tRNAscan-SE v2.0.5 program, and further manual adjustment was performed with BLATN and BLATX [36, 37]. The annotated chloroplast genome of *C*. *nipponicum* was submitted to GenBank under accession number MW248139. The genome map was illustrated by Organellar Genome DRAW (OGDRAW) [38]. The irScan and IRscope identified inverted repeat regions [39] for genomes with no information about IR annotations [40, 41]. Sequences of all protein-coding genes were used to analyze codon preference. Relative synonymous codon usage (RSCU) was calculated based on the following equation [42]:

RSCUij=Xij∑j=1njXijni

*X*_*ij*_ is the number of occurrences of the *j*th codon for the ith amino acid, and *n*_*i*_ is the number of alternative codons for the *i*th amino acid.

### Repeat sequence identification

Simple-sequence repeats (SSRs) in the C. nipponicum chloroplast genome were determined using MISA with the minimal repeat numbers set to 10, 5, 4, 3, 3, and 3 for mono-, di-, tri-, tetra-, penta-, and hexa-nucleotide, respectively [43]. REPuter was used to identify dispersed repeats, including forward, reverse, complement, and palindromic kinds of repeat sequences with a minimum size of 30 bp and hamming distance of 3 [44].

### Divergent hotspot identification

The MAFFT alignment [45], followed by DNASP [46] was performed to compare the chloroplast genome of *C*. *nipponicum* with following five *Cirsium* species: *C*. *japonicum*, *C*. *rhinoceros*, *C*. *eriophorum*, *C*. *vulgare*, and *C*. *arvense*. In order to identify variant divergence regions, the multiple sequence alignments were analyzed to calculate nucleotide diversity with window length 600 and step size 200 options.

### Phylogenetic analysis

Phylogenetic analyses were conducted using the *Cirsium* species with Cardueae tribe species and one *Gerbera jamesonii* as an outgroup. The multiple sequence alignment for 20 sequences listed in S1 Table was performed using MAFFT with 1.53 gap penalty and FFT-NS-2 default method [45]. PAUP and Modeltest were used for Bayesian inference [47, 48]. MrBayes [49] was implemented with 1,000,000 generations and 250,000 generations burn-in, as well as the maximum likelihood analysis to construct phylogenetic trees. IQ-tree was performed to estimate maximum likelihood with 1000 bootstrap replications [50].

## Results

### Chloroplast genome of *C*. *nipponicum*

We sequenced whole genomic paired-end data of *C*. *nipponicum* in 16,415,067,154 bp size. By trimming adapters and low-quality sequences, a total of 3,739,051,830 high-quality reads were used as GetOrganelle-1.7.1 [33] input for chloroplast genome assembly. Based on the seed reads identified from GetOrganelle with 88,093,650 bp in length and 577x in sequencing depth, chloroplast genomic DNA was assembled into a circular form (Fig 1B). The length of the assembled genome of *C*. *nipponicum* was 152,586 bp with quadripartite structures, consisting of a large single-copy (LSC) region of 83,520 bp and a small single-copy (SSC) region of 18,701 separated by two inverted repeats (IRa, IRb) of 25,191 bp each. The GC content of the *C*. *nipponicum* chloroplast genome was 37.69%, and that of LSC, SSC, and IRs regions were 35.83%, 37.49%, and 43.11%, respectively. LSC exhibited the lowest value of GC contents among the four regions of the chloroplast genome, and IR regions had the highest value.

Using PGA [35] and GeSeq [34] annotation tools, the chloroplast genome of *C*. *nipponicum* annotated 133 genes consisting of 8 rRNA genes, 37 tRNA genes, and 88 protein-coding genes (Table 1). In total of 133 genes, 18 genes including 7 tRNA genes (*trnI-CAU*, *trnL-CAA*, *trnV-GAC*, *trnI-GAU*, *trnA-UGC*, *trnR-ACG*, *trnN-GUU*), 4 rRNA genes (rrn4.5, rrn5, rrn16, rrn23), and 7 protein-coding genes (*rpl2*, *rpl23*, *rps7*, *rps12*, *ycf2*, *ycf15*, *ndhB*) were duplicated in IR regions. Also, 11 protein-coding genes (*rpl2*, *rpl16*, *rps12*, *rps16*, *rpoC1*, *atpF*, *ycf3*, *clpP*, *petB*, *petD*, *ndhA*, and *ndhB*) contained exons and introns. The small subunit ribosomal protein 12 (*rps12*) gene was trans-spliced, where the first exon was located in the LSC region and others in the IR regions.

**Table 1:** List of annotated genes in the *C*. *nipponicum* chloroplast genome.

Classification of Genes		Names of Genes	Number
RNA genes	Ribosomal RNAs	rrn4.5 (x 2), rrn5 (x 2), rrn16 (x 2), rrn23 (x 2)	8
	Transfer RNAs	*trnA-UGC* (x 2), *trnC-GCA*, *trnD-GUC*, *trnE-UUC*, *trnF-GAA*, *trnM-CAU*, *trnG-GCC*, *trnG-UCC*, *trnH-GUG*, *trnI-CAU* (x 2), *trnI-GAU* (x 2), *trnK-UUU*, *trnL-CAA* (x 2), *trnL-UAA*, *trnL-UAG*, *trnM-CAU*, *trnN-GUU* (x 2), *trnP-UGG*, *trnQ-UUG*, *trnR-ACG* (x 2), *trnR-UCU*, *trnS-GCU*, *trnS-GGA*, *trnS-UGA*, *trnT-GGU*, *trnT-UGU*, *trnV-GAC* (x 2), *trnV-UAC*, *trnW-CCA*, *trnY-GUA*	37
Protein Coding genes	Ribosomal proteins, large subunits	*rpl*14, *rpl*16, *rpl*2 (x 2), *rpl*20, *rpl*22, *rpl*23 (x 2), *rpl*32, *rpl*33, *rpl*36	11
	Ribosomal proteins, small subunit	*rps*11, *rps*12 (x 2), *rps*14, *rps*15, *rps*16, *rps*18, *rps*19, *rps*2, *rps*3, *rps*4, *rps*7 (x 2), *rps*8	14
	RNA polymerases	*rpoA*, *rpoB*, *rpoC*1, *rpoC*2	4
	Photosystem 1	*psaA*, *psaB*, *psaC*, *psaI*, *psaJ*	5
	Photosystem 2	*psbA*, *psbB*, *psbC*, *psbD*, *psbE*, *psbF*, *psbH*, *psbI*, *psbJ*, *psbK*, *psbL*, *psbM*, *psbT*, *psbZ*	14
	Cytochrome b6/f complex	*petA*, *petB*, *petD*, *petG*, *petL*, *petN*	6
	ATP synthase	*atpA*, *atpB*, *atpE*, *atpF*, *atpH*, *atpI*	6
	NADH dehydrogenase	*ndhA*, *ndhB* (x 2), *ndhC*, *ndhD*, *ndhE*, *ndhF*, *ndhG*, *ndhH*, *ndhI*, *ndhJ*, *ndhK*	12
	Rubisco	*rbcL*	1
	*clpP*, *matK*	*clpP*, *matK*	2
	Hypothetical chloroplast reading frames (*ycf*)	*ycf*1 (x 2), *ycf*15 (x 2), *ycf*2 (x 2), *ycf*3, *ycf*4	8
	Other genes	*accD*, *ccsA*, *cemA*, *infA*, *pbf*1	5
Total			133

To distinguish the *C*. *nipponicum* chloroplast genome within other *Cirsium* species, we compared five well-known chloroplast genomes from NCBI RefSeq Sequences and reassigned quadripartite structures: *C*. *arvense* (NC_03695.1), *C*. *vulgare* (NC_036967.1), *C*. *eriophorum* (NC_036966.1), *C*. *rhinoceros* (NC_044423.1), and *C*. *japonicum* var. spinosissimum (NC_050046.1). All of these species, except for *C*. *eriophorum*, were found in Korea. *C*. *vulgare* and *C*. *arvense* were exotic species distributed worldwide including Russia, China, and Japan, and the remaining two, *C*. *rhinoceros* and *C*. *japonicum*, were endemic to Korea. As a result of basic statistics from comparing each chloroplast genome, the *C*. *nipponicum* chloroplast genome showed the lowest GC content in the whole chloroplast genome among six *Cirsium* species, whereas the GC content in the SSC region showed the highest value (Table 2).

**Table 2:** Basic features of six *Cirsium* chloroplast genomes.

Species	*C*. *nipponicum*	*C*. *japonicum*	*C*. *rhinoceros*	*C*. *eriophorum*	*C*. *vulgare*	*C*. *arvense*
Total length (bp)	152,586	152,342	152,576	152,557	152,567	152,855
IR length (bp)	25,191	25,191	25,806	25,176	25,076	25,182
LSC length (bp)	83,502	83,254	83,662	83,486	83,738	83,859
SSC length (bp)	18,701	18,706	18,742	18,719	18,677	18,632
Total gene number	133	127	133	133	133	133
CDS number	88	83	88	88	88	88
rRNA number	8	8	8	8	8	8
tRNA number	37	36	37	38	37	37
GC %	37.69	37.72	37.71	37.70	37.70	37.71
LSC GC %	35.83	35.88	35.84	35.85	35.81	35.87
SSC GC %	37.49	31.34	31.37	31.38	31.39	31.37
IR GC %	43.11	43.11	43.20	43.11	43.20	43.11
GenBank accession	.	NC_050046.1	NC_044423.1	NC_036966.1	NC_036967.1	NC_036965.1

### Expansion and contraction of IR regions

**Fig 2:**
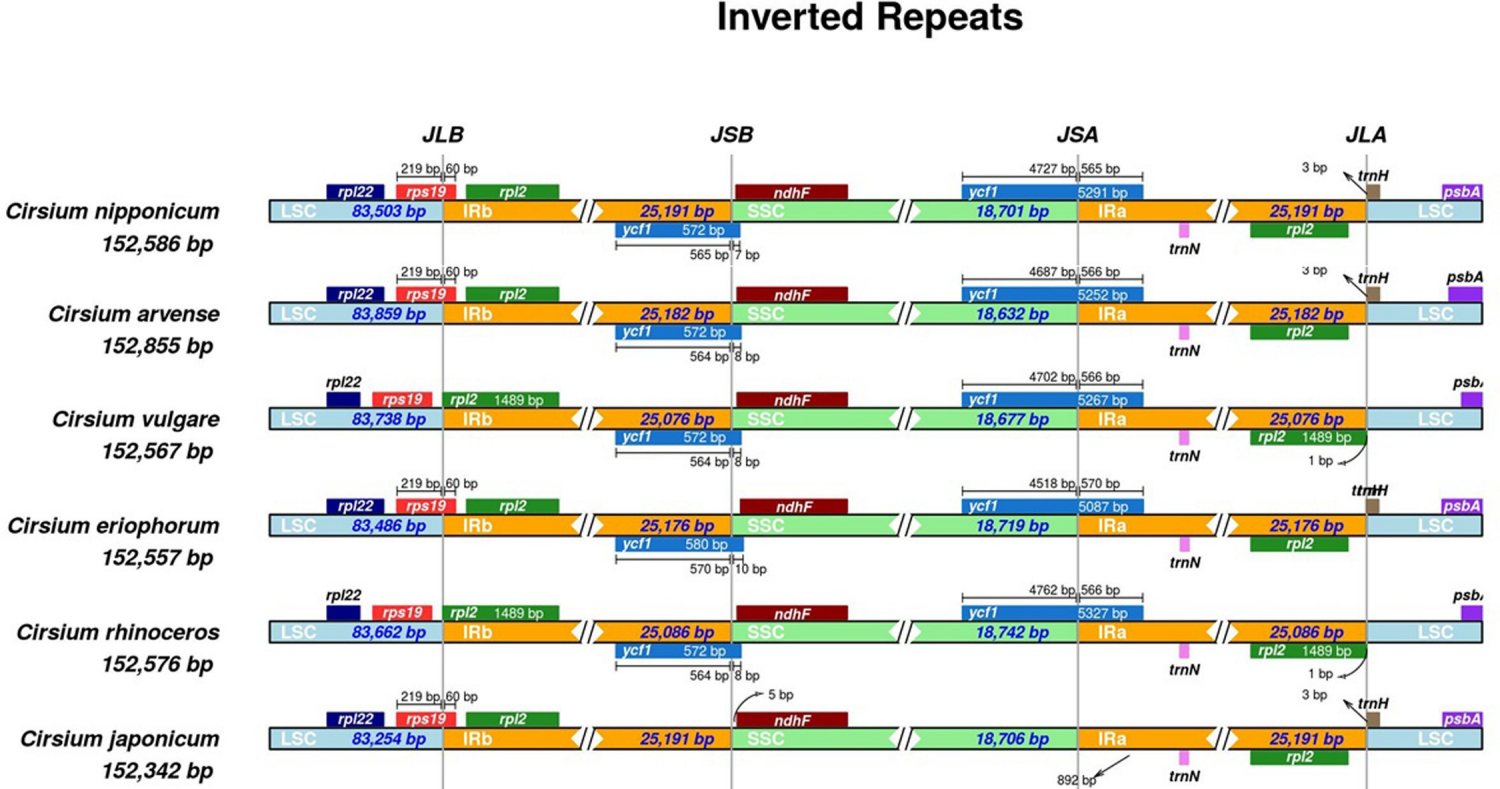
Comparison of the IR regions and the junctions of LSC, IR, and SSC regions among chloroplast genomes of six *Cirsium*. *C*. *arvnes*, *C*. *vulgare*, *C*. *eriophorum*, *C*. *rhinoceros*, *C*. *japonicum* have NC_036965.1, NC_036967.1, NC_036966.1, NC_044423.1, NC_050046 accession numbers respectively.

Many studies have identified variations in the length of chloroplast genomes when comparing IR regions, including boundary junctions within the same genus species. Considering that the chloroplast genome is regarded as the most conserved region, the appearance of expansion and contraction in IR regions could be a part of the genome evolution. Thus, we performed IRscope [41] with six *Cirsium* species to investigate the differences in IR regions (Fig 2). As a result, the *rps19* gene showed across a junction between LSC and IR regions in *C*. *nipponicum*, *C*. *arvense*, *C*. *eriophorum*, and *C*. *japonicum*. On the other hand, the *rpl2* gene was across the junction in *C*. *vulgare* and *C*. *rhinoceros*. The gene pattern around the IR junction of *C*. *nipponicum* was similar to that of *C*. *arvense* and *C*. *eriophorum*. Subsequently, multiple sequence alignment using chloroplast genome based on *C*. *nipponicum* IR regions revealed four deletions-two in *ycf2* gene, one in *trnI-GAU* gene, and one in the intergenic region between rrn5 and *trnR-ACG*-and two insertions in *ycf2* gene and the same intergenic region as deletion (S2 Table).

### Codon preference analysis

**Fig 3:**
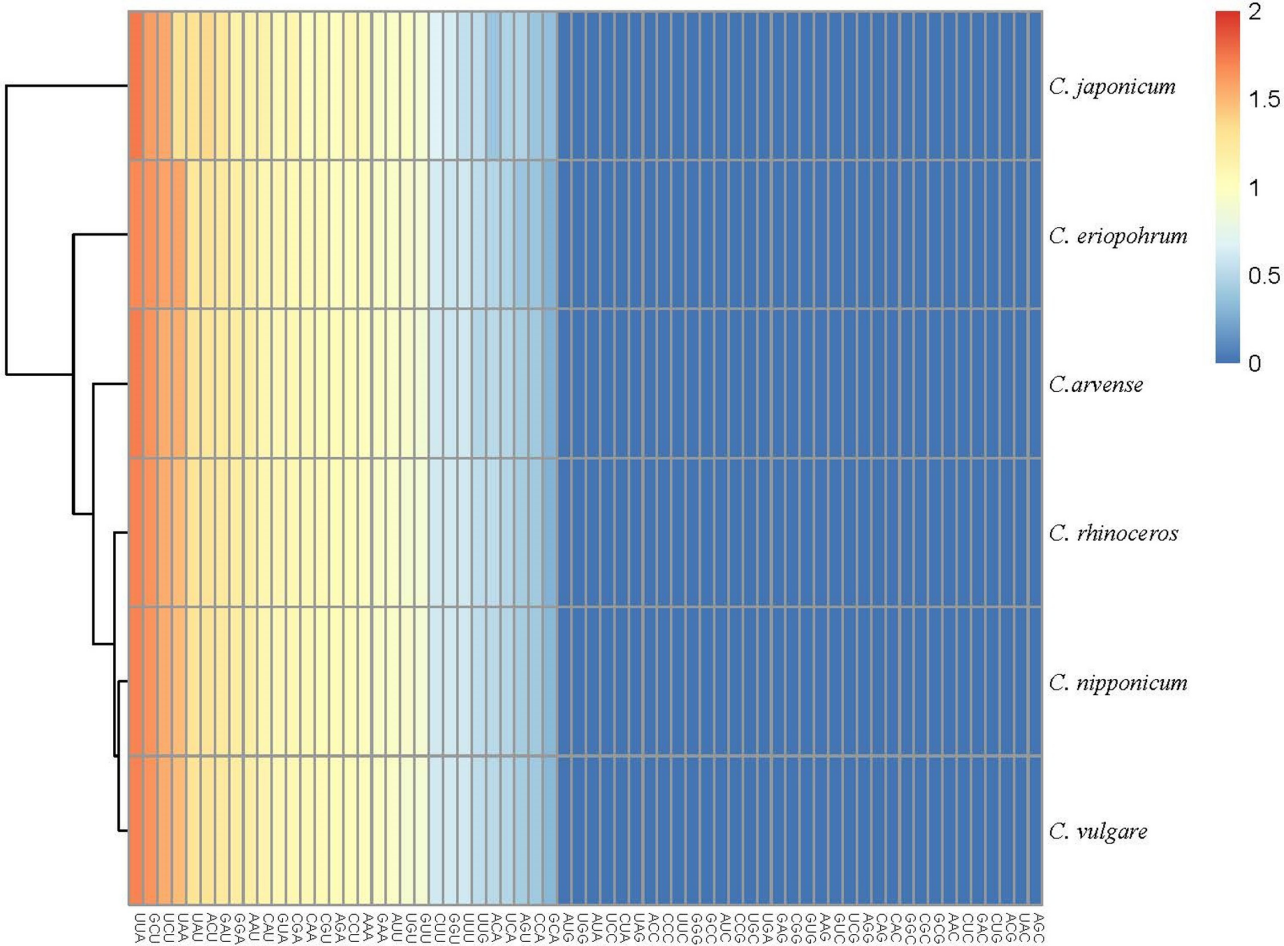
Heat map of relative synonymous codon usage values of chloroplast protein coding genes among six *Cirsium* species.

We analyzed the frequency of codon usage using the protein-coding genes of *C*. *nipponicum*, including the other five *Cirsium* species. As a result, isoleucine and leucine were the most abundant amino acids (10. 86%, 10.63%), while cysteine was the least encoded (1.12%) in *C*. *nipponicum* (S3 Table). The percentage of the amino acids in the other five *Cirsium* species showed the same pattern as *C*. *nipponicum* (S1 Fig). Furthermore, all amino acids were found in the six *Cirsium* chloroplast genomes and exhibited codon preference except methionine and tryptophan. As we calculate the relative synonymous codon usage (RSCU) of *C*. *nipponicum* to measure the extent of codon bias, there were 30 codons with high preference (RSCU > 1) and 32 codons with low preference (RSCU < 1) out of 64 codons encoded 20 amino acids. The highest value of the RSCU codon was UUA (1.80–1.83), and the lowest codon was AGC (0.35–0.38) in all chloroplast. The patterns of RSCU values were similar to *C*. *vulgare* (Fig 3). Twenty-nine codons with RSCU values greater than 1 were codons ending with A or U, whereas 29 out of 32 codons with RSCU values less than 1 were codons ending with G or C.

### Repeat sequence analysis

**Fig 4:**
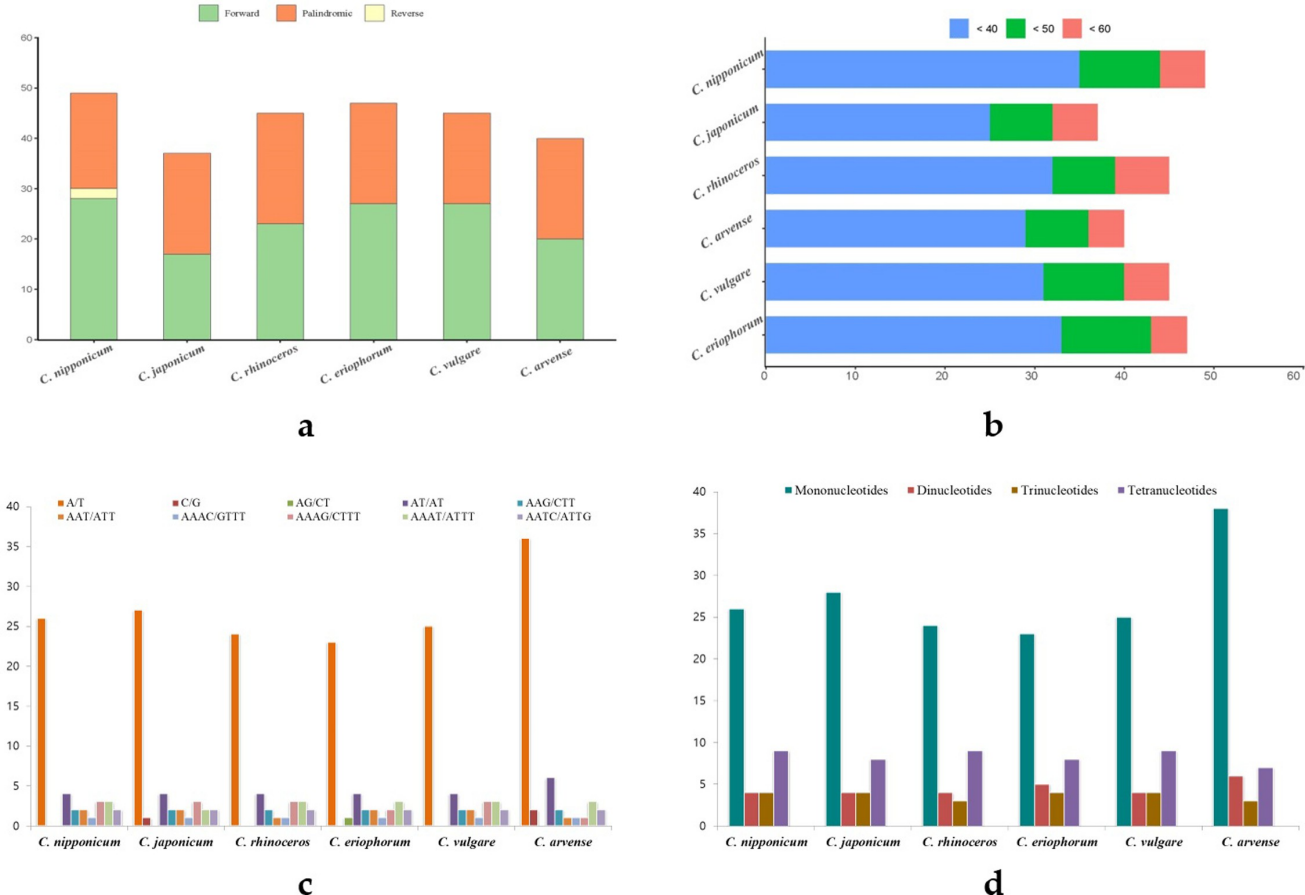
The number and type of repeats in six *Cirsium* species. a. Frequency of three types dispersed repeats; b. Frequency of dispersed repeats by length; c. Frequency of simple sequence repeats (SSRs) motifs in different types; d. Frequency of four SSRs types.

Repeat elements have essential roles in characterizing genomes with particular perspectives. Especially in terms of conservativeness in the chloroplast genome, it can be helpful in species identifications. We identified dispersed repeats in six *Cirsium* species using REPuter (Kurtz et al., 2001) software (Fig 4A and 4B). The dispersed repeats were detected in three types of repeats (forward, reverse, palindromic) and ranged from 30 to 58 bp in length. Among these species, *C*. *nipponicum* contained the largest number of repeats and only carried a reverse type of repeats. The total number of dispersed repeats in *C*. *nipponicum* was 49, consisting of 28 forward, two reverse, and 19 palindromic repeat sequences. These repeats were located in various regions: three non-coding genes, 24 coding genes, 18 intergenic, and four intergenic spacers (IGSs) (S4 Table).

In addition to dispersed repeats, simple sequence repeats (SSRs), also known as microsatellites, were investigated using the MISA program [43]. There were 40 to 54 SSRs in *Cirsium* species, and mono-, di-, tri-, and tetra-nucleotide were detected in all *Cirsium* chloroplast genomes (Fig 4C and 4D). Most SSRs consisted of mono-nucleotide with the A/T motif, but the C/G motif was presented only in *C*. *arvense* and *C*. *japonicuum*. Moreover, *C*. *nipponicum* showed 43 SSRs with 26 mono-, 4 di-, 4 tri-, and 9 tetra-nucleotides. They were located in LSC regions (74%) and SSC regions (21%), and only a few in IR regions (5%) (S5 Table).

### Divergence of hotspot regions

**Fig 5:**
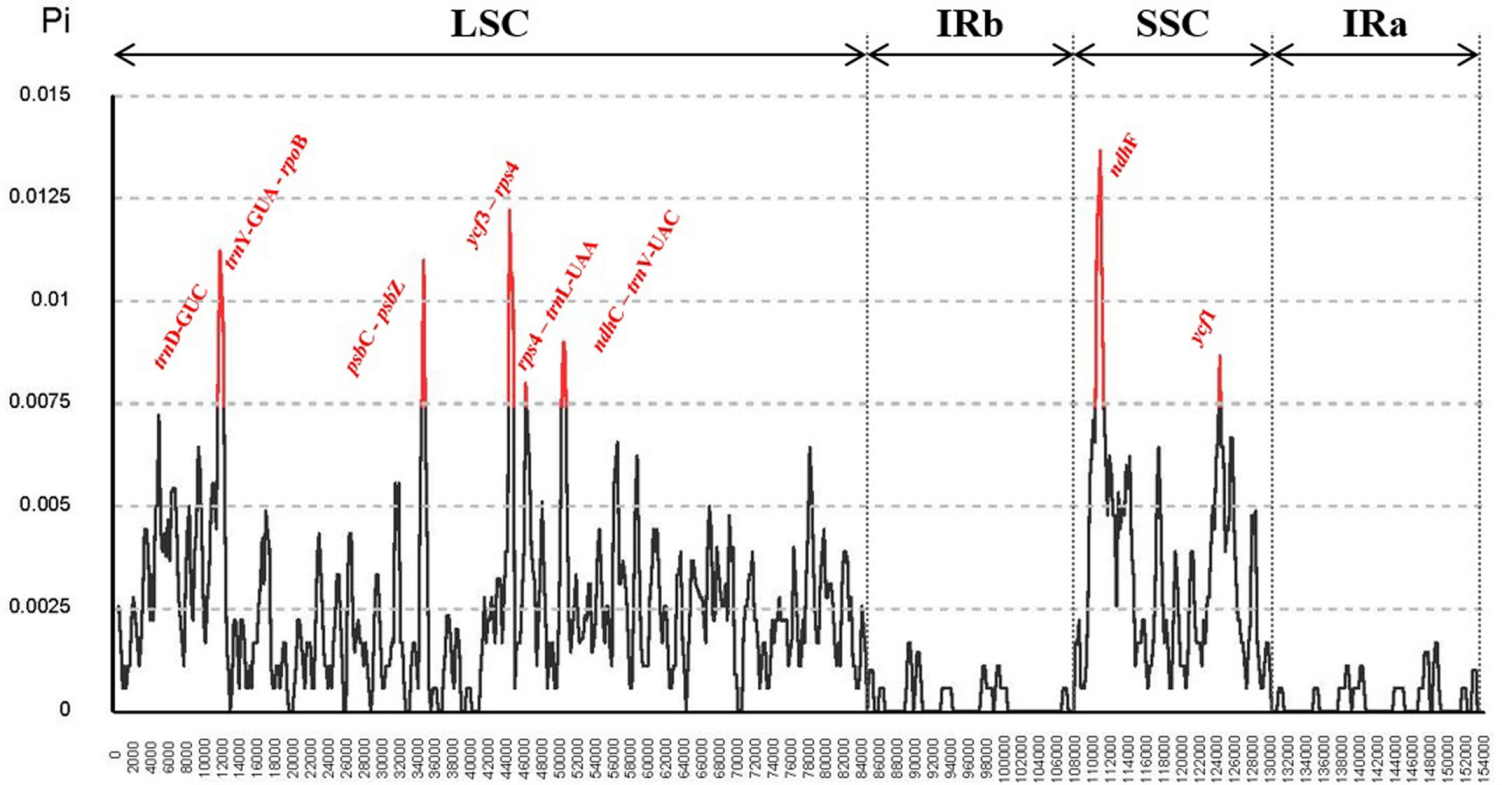
Sliding window of nucleotide diversity from the alignment of six *Cirsium* plastomes.

Highly variable regions in chloroplast genomes have been widely used in species identification studies. Since only morphological characteristics in *Cirsium* limit the distinction between each species, we performed multiple sequence alignment using six *Cirsium* species to find highly variable regions. As a result, there were 833 polymorphic sites, and the nucleotide diversity was calculated over the whole chloroplast genome (Fig 5). Among six *Cirsium* species, Pi values ranged from 0 to 0.01367 with an average of 0.00195. The highly variable regions that contain polymorphic sites were considered when Pi values were greater than 0.00743. The number of regions exceeding a given threshold was eight, with highly variable sites only in LSC and SSC regions (S6 Table). Three of the eight highly variable regions were located in coding sequences (*trnD-GUC*, *ndhF*, and *ycf1*), and the remaining five regions were spanned intergenic regions. Moreover, 18 specific variations were identified, mainly focusing on distinguishing *C*. *nipponicum* from other species (S7 Table). The regions that contained these specific substitutions were also in LSC and SSC regions.

### Phylogenetic analysis and species resolution

**Fig 6:**
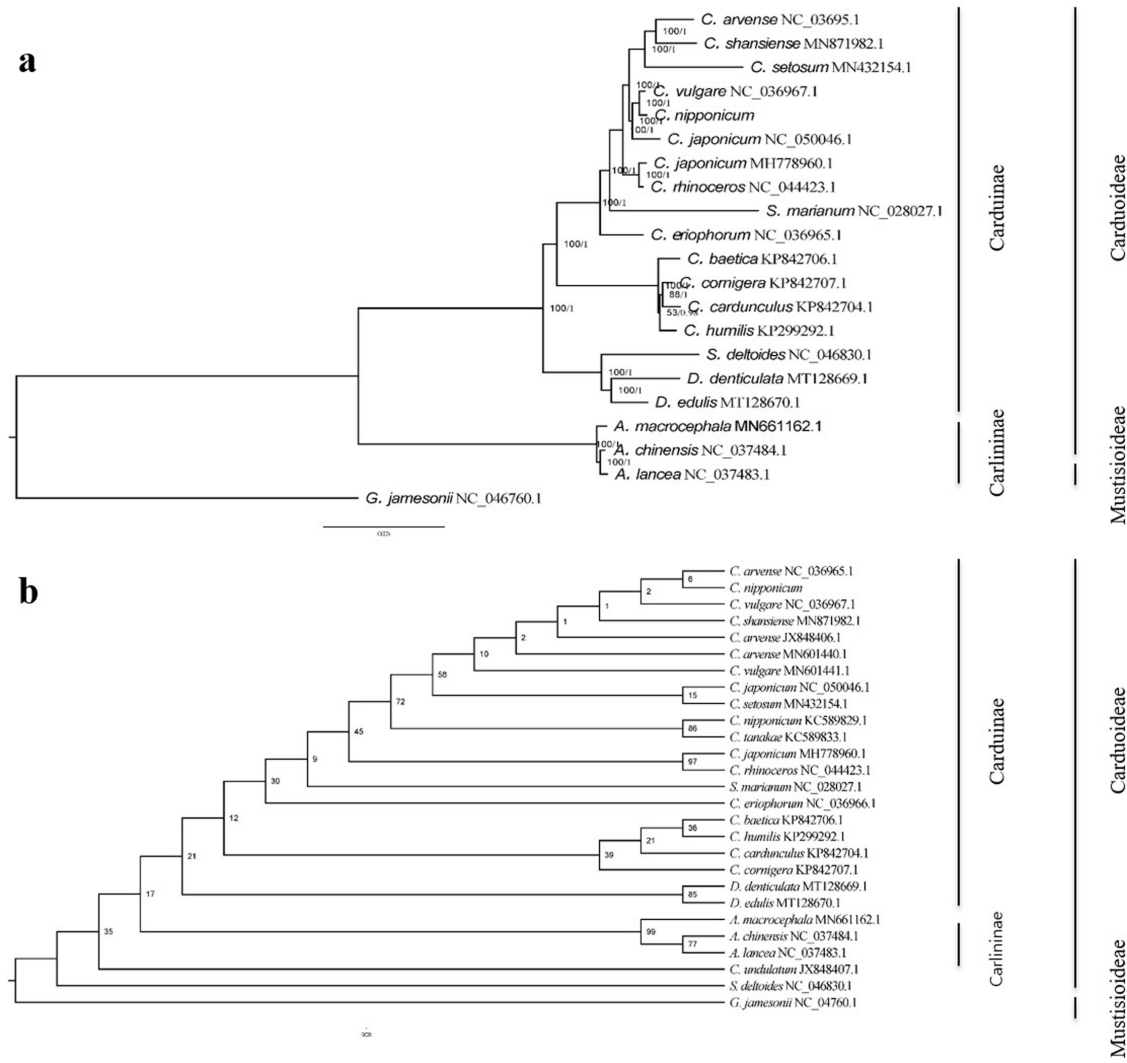
Phylogenetic trees based on the whole chloroplast genomes and the *rbcL*. (a) Phylogenetic relationship based on whole chloroplast genomes inferred by maximum likelihood (ML) with numbers beside the nodes representing the ML bootstrap values and Bayesian inference posterior probabilities; (b) Phylogenetic relationship based on the *rbcL* inferred by ML with numbers besides the nodes representing the ML bootstrap values.

For a better understanding of the phylogenetic relationship among *C*. *nipponicum* and other species across tribes, phylogenetic analysis with two methods, Bayesian inference (BI) and maximum likelihood (ML), was conducted with *Gerbera jamesonii* as an outgroup. First, we achieved 20 complete chloroplast genomes from NCBI and then estimated the substitution model, known as the DNA sequence evolution model. Based on the best substitution models, TVM+F+I+G4 in the ML method and GTR+I+G in the BI method were applied to construct phylogenetic trees, and both results showed the same topology structure (Fig 6A). In the subtribe level, three Carlininae species and 17 Carduinae species were separated into each clade, and *C*. *vulgare* was the closest to *C*. *nipponicum*. In addition, we used *matK* and *rbcL* sequences to get more information about relationships between species and the resolution of speciation (Figs 6B and S2 and S3). Phylogenetic trees constructed by *matK* gene sequences showed similar results to complete chloroplast genome trees. The *matK* gene trees based on BI and ML methods had the same patterns of topology structure, and species were clustered by subtribe and genus levels. However, *Cirsium* species were split with low bootstrap values in the ML tree. When using *rbcL* gene sequences, we obtained six more sequences, 4 Sanger and 2 Illumina sequencing platforms, from the NCBI database to find the relationship of *C*. *nipponicum* sampled from Ulleung Island with others, especially with other Korean *Cirsium* and *C*. *nipponicum* KC589829.1, distributed in Japan. As a result, two of the *rbcL* trees had similar but low bootstrap values, especially low posterior probabilities around *Cirsium* species (S3 Fig). Moreover, Japanese *C*. *nipponicum* KNC589829.1 was close to Japanese *C*. *tanakae* and Korean *C*. *japonicum*, not to Ulleung Island *C*. *nipponicum*; however, *C*. *nipponicum* from Ulleung Island was still close to *C*. *vulgare* and *C*. *arvense*. The trees made by three sequence types revealed that *C*. *nipponicum* was far from *C*. *japonicum* and *C*. *rhinoceros* compared to *C*. *vulgare* and *C*. *arvense* in phylogenetic relationships.

## Discussion

Although the advances in high-throughput sequencing technologies has facilitated rapid progress in the field of genomics as well as chloroplast genetics [23], limited chloroplast genomes of *Cirsium* species were available. Herein, we present the complete chloroplast genome of *C*. *nipponicum* for the first time and provide convincing evidence for the distinctive origin and evolution of *C*. *nipponicum* by analyzing genome structure and phylogenetic relationships among *Cirsium* species. As a result, GC contents in the IR regions of six *Cirsium* species were higher than both LSC and SSC regions, indicating the presence of rRNA [51, 52]. Besides, when considering that the GC content of the SSC region in *C*. *nipponicum* is relatively higher than others, GC-biased gene conversion (gBGC) related to intraplastomic recombination could be proposed as another cause of GC content pattern [53–55]. These GC content patterns and repeat elements are helpful in identifying speciation because of their polymorphism [56]. Identifying speciation based on a molecular marker such as a barcode system is important to the efficiency of species protection and management [29]. For DNA primer candidates, we found some repeats in several genes, including *ndhA*, *ycf1*, and near rRNA and IGS (S5 and S6 Tables). Furthermore, as these SSRs and dispersed repeats affect the genetic investigations such as population or phylogenetic relationship [15, 57], this study suggests its applicability to the evolution mechanism of *Cirsium*, especially in genetic structures of chloroplast genomes.

The codon usage bias is commonly observed in genomes of all organisms, including plants, such that understanding the evolutionary significance of its phenomenon was a common interest among biologists. The usage of synonymous codons for amino acids is not random, but it has bias [58], which is related to highly expressed genes and even plays a role in the evolution of chloroplast genomes [59, 60]. Since the chloroplast genome of plants is well-known to have the codon usage bias, the analysis of RSCU in the chloroplast of *C*. *nipponicum* can help understand genetic features and evolutionary process [61, 62]. Our results showed that the patterns of RSCU in *C*. *nipponicum* were more similar to *C*. *vulgare* than *C*. *rhinoceros* and *C*. *japonicum* (Fig 3). Hence, the preference for synonymous codons may imply a part of chloroplast genome evolution in *Cirsium* species.

We used five whole chloroplast genomes of *Cirsium* species available in the NCBI RefSeq database, considering the data validation and updates to reflect current knowledge, to perform comparative analyses. Compared with a previous study of three *Carduus* species that belong to the same subtribe as *Cirsium*, which reported nucleotide diversity with an average of 0.003442 and a peak of 0.0171 [63], our study showed that *Cirsium* species are more stable and conservative than *Carduus* species. Furthermore, the variation analysis results were consistent with the general feature, such that IR regions in the chloroplast of angiosperm were the most conserved region (S6 Table). Interestingly, when comparing the IR regions, *C*. *niponnicum* was close to *C*. *japonicum*, whereas the whole chloroplast genome was close to *C*. *vulgare*. Despite that expansion and contraction in IR regions are essential to the evolutionary process in chloroplast genome size [64, 65], variation in whole regions was more related to speciation within *Cirsium* species than in IR region. Recently, many researchers have used barcode systems for species separation using meta-barcode or universal mini-barcodes called *matK* and *rbcL* [66]. However, our constructed phylogenetic trees with *matK* and *rbcL* genes separately presented a low bootstrap value of ML and probability of BI, which indicate an unreliable topology, especially in *matK* (S2A Fig). Thus, we believe that phylogenetic trees using mini-barcodes could not be an appropriate method for speciation within *Cirsium* species.

As *C*. *nipponicum* is predominantly located on Ulleung Island, we initially thought it could be evolutionary similar to those close to the mainland or Japan, just like other plants growing on Ulleung Island. Ulleung Island is located about 137 km off the east coast of the Korean peninsula and was formed approximately 2 million years ago (Mya) [67, 68]. It is known to have about 600 taxa of vascular plants on Ulleung Island and is suggested to be derived and evolved from a founder population from the land close to the island, a mode of speciation known as anagenetic speciation [69]. However, our results showed that *C*. *nipponicum* was not grouped with two Korean species, *C*. *rhinoceros* and *C*. *japonicum*, or two Japanese species, *C*. *nipponicum* and *C*. *tanakae* (Figs 6 and S3). Moreover, *C*. *nipponicum* from Ulleung Island was more closely related to *C*. *vulgare* than others. The patterns of morphological characters in *C*. *nipponicum* are also distinct from other *Cirsium* species, such as *C*. *japonicum* and *C*. *rhinoceros* [1]. Additionally, the leaf shape of *C*. *nipponicum* is morphologically most similar to that of *C*. *vulgare* among the other *Cirsium* species around Ulleung Island (Fig 1C–1F). Therefore, *C*. *nipponicum* in Ulleung Island may not be originated from endemic species of Japan or Korea, but it may instead be derived from Russia [70], given the distribution of *C*. *vulgare* that is not distributed in Korea.

Based on the fact that the *Cirsium* species is known as a cosmopolitan [71], the probability of its dispersal to Ulleung Island can be inferred in several ways. One of the most effective methods to disperse the seeds of the family Asteraceae has been suggested as wind [72]. Although westerly winds are the dominant winds in Ulleung Island, dispersing by wind may be limited considering that there is no *C*. *vulgare* in the Korean peninsula, which is registered as invasive species by the Korean government [73]. Ocean currents are another possibility of dispersing, suggesting that dispersal of *Fangus* via floating masses from the north and south to Ulleung Island is possible [69]. Lastly, the dispersal of migratory birds traveling to Ulleung Island is another possibility. It has been reported that transporting seeds by birds may occur in Northeast China, Far East Russia, and Southern Korea and Japan [69] to the extent that reports of waterfowls passing through Ulleung Island were identified [74]. Some of these waterfowls were regarded as important vectors of exotic plant species [75]. Thus, endozoochory by waterfowls can be suggested as a factor explaining the dispersal of *C*. *nipponicum* on Ulleung Island. This study suggested that *C*. *nipponicum* of Ulleung Island originated from *Cirsum* other than Korean or Japansese endemic Cirsium, and has been adapted to the Ulleung Island environment.

## Supporting information

S1 Fig Amino acid percentages of six *Cirsium* chloroplast genomes. (TIF)

S2 Fig Phylogenetic tree based on the *matK*. (a) Phylogenetic tree based on the *matK* inferred by Maximum likelihood (ML) with number beside the nodes representing the ML bootstrap values. (b) Phylogenetic tree based on the *matK* inferred by Bayesian inference (BI) with numbers beside the nodes representing the BI posterior probabilities.
(TIF)

S3 Fig Phylogenetic tree based on the *rbcL* inferred by BI. Numbers beside the nodes represent the BI posterior probabilities.
(TIF)

S1 Table The list of sequences used in phylogenetic analysis. (XLSX)

S2 Table Result of multiple sequence alignment in IR regions based on *C*. *nipponicum*. Ins and Del mean insertion and deletion respectively.
(XLSX)

S3 Table Codon frequency of all protein coding genes in chloroplast genome of *C*. *nipponicum*. (XLSX)

S4 Table List of disperse repeats in *C*. *nipponicum*. F, P, R mean the direction of repeats, forward, palindromic, reverse respectively.
(XLSX)

S5 Table The list of simple sequences repeats in *C*. *nipponicum*. (XLSX)

S6 Table Highly variable regions detected in *C*. *nipponicum*. (XLSX)

S7 Table *C*. *nipponicum* specific variable regions. (XLSX)
