# Supplementary figures and images for: Distinctive origin and evolution of endemic thistle of Korean volcanic island: Structural organization and phylogenetic relationships with complete chloroplast genome
Source: PLoS One. 2023 Mar 13;18(3):e0277471. doi: 10.1371/journal.pone.0277471 (PMC10010555; doi:10.1371/journal.pone.0277471)

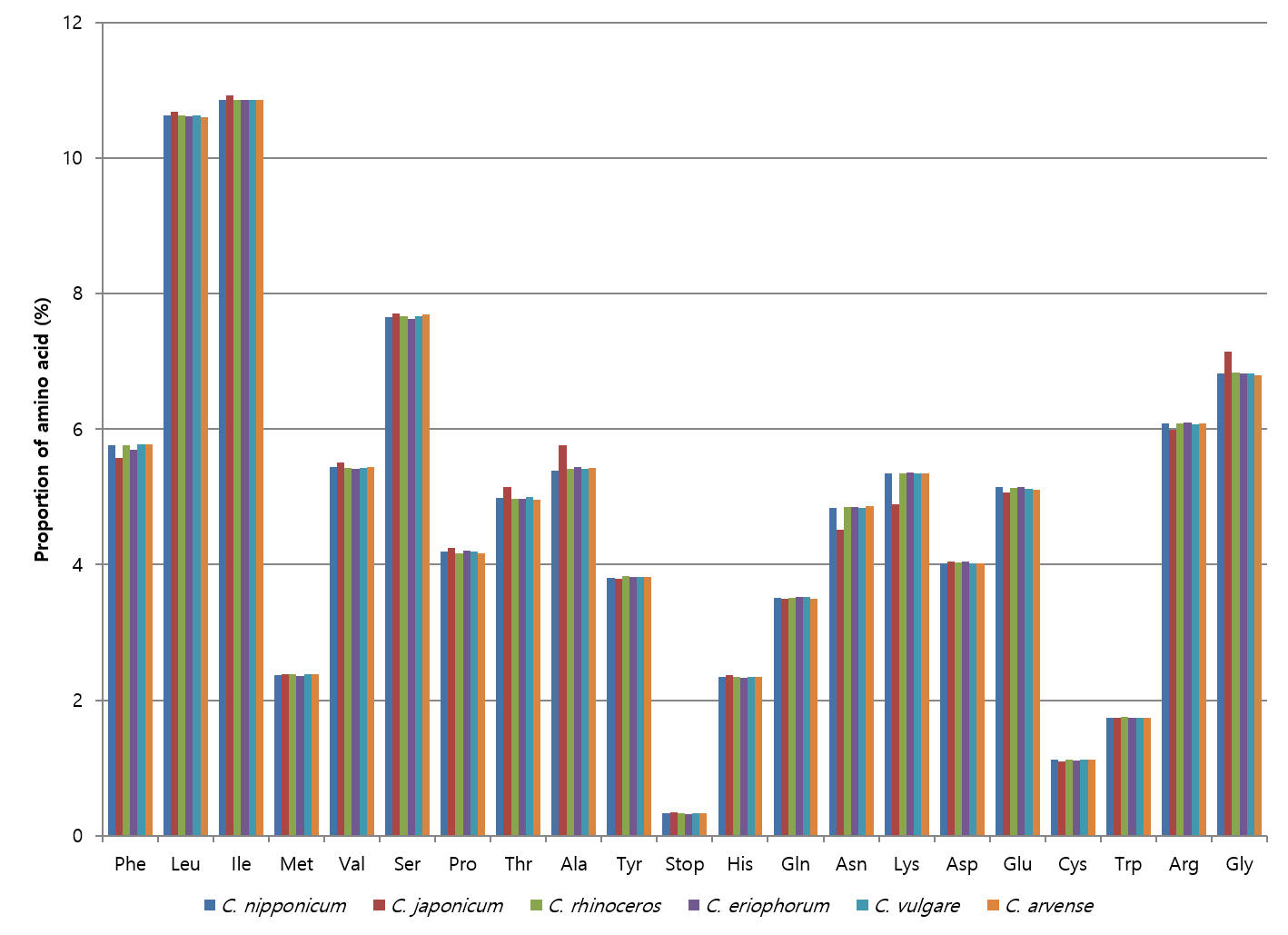

Supplement: S1 Fig — (TIF) [file pone.0277471.s001.tif]

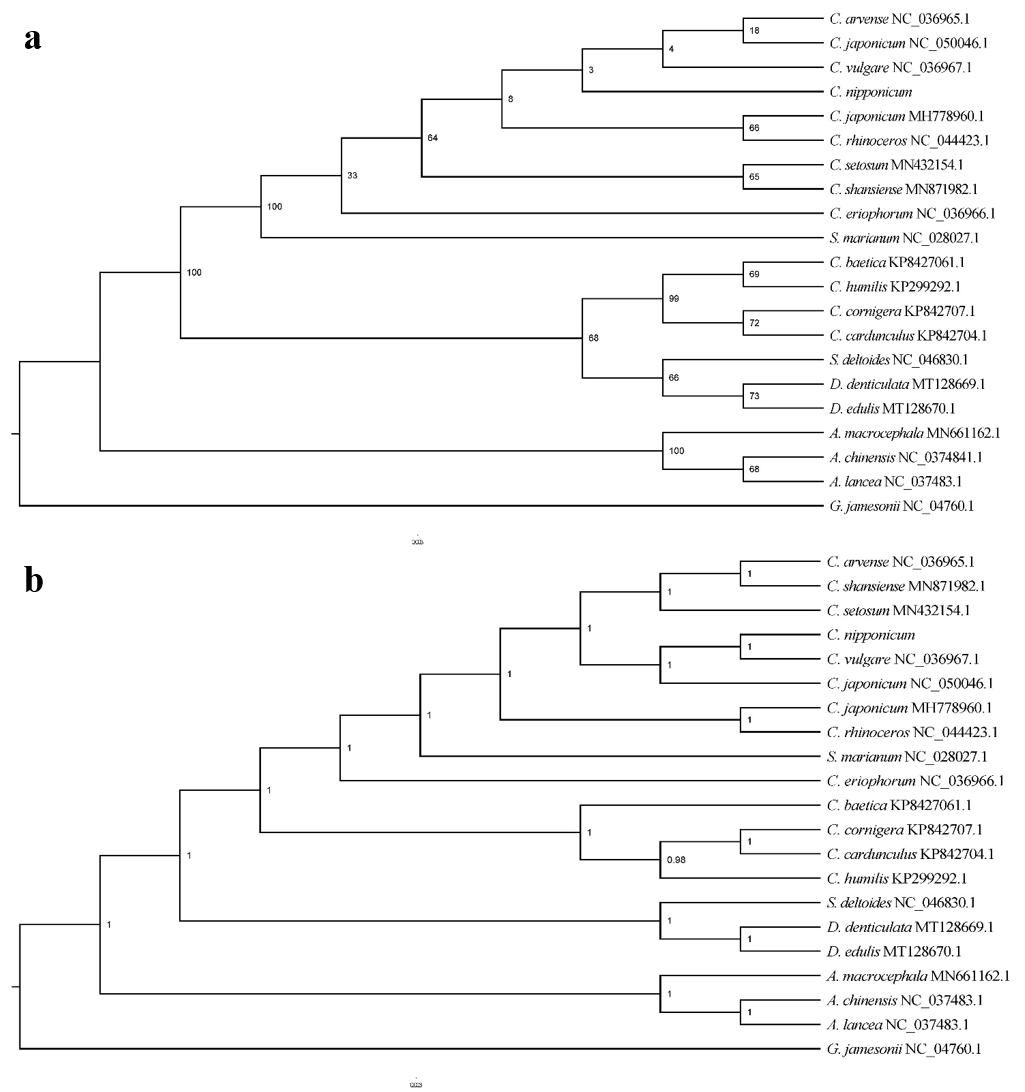

Supplement: S2 Fig — (a) Phylogenetic tree based on the matK inferred by Maximum likelihood (ML) with number beside the nodes representing the ML bootstrap values. (b) Phylogenetic tree based on the matK inferred by Bayesian inference (BI) with numbers beside the nodes representing the BI posterior probabilities. (TIF) [file pone.0277471.s002.tif]

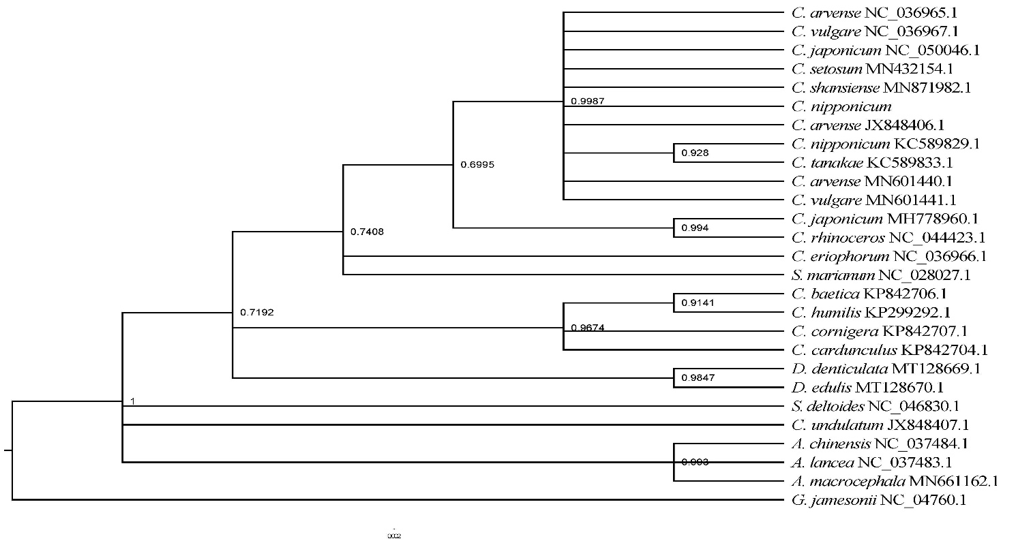

Supplement: S3 Fig — Numbers beside the nodes represent the BI posterior probabilities. (TIF) [file pone.0277471.s003.tif]
